# Immunostimulatory activity of lifespan-extending agents

**DOI:** 10.18632/aging.100619

**Published:** 2013-12-05

**Authors:** José Manuel Bravo-San Pedro, Laura Senovilla

**Affiliations:** ^1^ INSERM U848; Villejuif, France; ^2^ Equipe 11 labellisée par la Ligue Nationale contre le Cancer, Centre de Recherche des Cordeliers; Paris, France; ^3^ Gustave Roussy; Villejuif, France; ^4^ INSERM U1015; Villejuif, France

**Keywords:** ATG5, everolimus, mTOR, sirolimus, tacrolimus, temsirolimus

## Abstract

During the past two decades, several interventions have been shown to increase the healthy lifespan of model organisms as evolutionarily distant from each other as yeast, worms, flies and mammals. These anti-aging maneuvers include (but are not limited to) cycles of caloric restriction, physical exercise as well as the administration of multiple, chemically unrelated agents, such as resveratrol, spermidine and various rapamycin-like compounds collectively known as rapalogs. Most, if not all, lifespan-extending agents promote macroautophagy (hereafter referred to as autophagy), an evolutionarily old mechanism that contributes to the maintenance of intracellular homeostasis and plays a critical role in the adaptive response of cells to stress. In line with this notion, the activation of autophagy appears to mediate significant anti-ageing effects in several organisms, including mice. Here, we focus on rapalogs to discuss the possibility that part of the beneficial activity of lifespan-extending agents stems from their ability to exert immunostimulatory effects. Accumulating evidence indicates indeed that the immune system can recognize and eliminate not only cells that are prone to undergo malignant transformation, but also senescent cells, thus playing a significant role in the control of organismal aging. In addition, it has recently become clear that rapamycin and other rapalogs, which for a long time have been viewed (and used in the clinic) as pure immunosuppressants, can mediate robust immunostimulatory functions, at least in some circumstances.

The hypothesis that organismal aging might be slowed down, and hence the appearance of aging-associated disorders delayed, has been the subject of intense investigation throughout the past two decades [1, 2]. In this context, several interventions have been demonstrated to significantly extend the healthy lifespan of model organisms as distant from each other on the evolution scale as yeast, worms, flies and mammals [3-6]. For illustrative purposes, such interventions can be classified into two large groups: (1) lifestyle modifications and (2) pharmacological/genetic maneuvers. The former include cycles of caloric restriction as well as a regular physical activity. These are actually known to extend the healthy lifespan of humans since a long time, although the molecular mechanisms underlying this phenomenon have only recently begun to emerge [7-10]. The latter encompass the administration of an increasingly wide panel of chemically unrelated molecules, including (but not limited to) resveratrol (a polyphenol found in grapes and red wine), spermidine (a polyamine that is abundant in grapefruits and soybeans), rapamycin (a macrolide originally isolated from the Easter Island micro-organism *Streptomyces hygroscopicus*) and multiple rapamycin-like compounds that are collectively known as rapalogs [11-15]. Most, if not all, these interventions share the ability to promote macroautophagy (hereafter referred to as autophagy), a mechanism for the lysosomal degradation of super-fluous, damaged or ectopic intracellular constituents [16, 17]. Moreover, the beneficial effects of both lifestyle modifications and pharmacological/genetic maneuvers have been shown to depend on an intact autophagic machinery, at least in some models [18-21]. In line with this notion, the moderate overexpression of one essential mediator of autophagy (i.e., ATG5) at the whole body level has recently been shown to extend the median lifespan of mice by approximately 17% [22]. Conversely, the genetic inhibition of autophagy-relevant proteins such as Beclin 1 (ATG6), ATG7 and ATG12 reportedly mediates a negative effect on the healthy lifespan of model organisms including *Caenorhabditis elegans* [23]. As a matter of fact, baseline levels of autophagy play a major role in the maintenance of intracellular (and hence organismal) homeostasis, hence mediating a robust oncosuppressive activity [24-26]. In addition, autophagy orchestrates the adaptive response of cells to multiple adverse conditions, including nutritional, physical and chemical cues [27]. It is therefore not surprising that autophagy might increase the organismal fitness and hence delay aging [28].

Nonetheless, the precise mechanisms whereby specific changes in lifestyle as well as selected chemicals or genetic manipulations delay aging (at least in model organisms) have not yet been fully elucidated. Thus, the efficacy of some anti-aging interventions may rely on mechanisms other than the upregulation of the autophagic flux. The immune system stands out as a good candidate for a part in this process, based on at least two lines of evidence: (1) autophagy plays a major role not only in the activation of innate responses against intracellular pathogens at the cell-autonomous level [29, 30], but also in the elicitation of adaptive immune responses based on the interaction between antigen-presenting cells and antigen-specific CD4^+^ and CD8^+^ T lymphocytes [31-33]; and (2) the immune system has been shown to recognize and eliminate not only cells that are prone to undergoing malignant transformation, but also senescent cells, thus contributing to the control of organismal aging [34, 35]. Interestingly, however, rapamycin and other rapalogs have long been known (and currently employed in the clinic) for their capacity to mediate robust immunosuppressive effects [36-38]. Indeed, rapamycin (which is also known as sirolimus) has first been approved by the US Food and Drug Administration (FDA) in 1999 for use in combination with ciclosporin and corticosteroids to prevent acute organ rejection in patients receiving kidney transplants [39, 40]. As it stands, however, the immunosuppressive potential of rapamycin and multiple rapalogs in humans has never been properly tested, as the clinical trials performed to date invariably employed as a control condition the gold-standard immunosuppressive regimens available [41]. In addition, accumulating preclinical and clinical evidence indicates that, at odds with immuno-suppressants that operate by inhibiting calcineurin, such as tacrolimus, rapamycin and other rapalogs might exert a significant immunostimulatory activity, at least under some circumstances.

This hypothesis first originated from the observation that the recipients of solid organs maintained on rapamycin-based regimens manifested a reduced incidence of various tumors, notably lymphoma, as compared to patients subjected to organ transplantation and treated with conventional immunosuppressants such as corticosteroids, ciclosporin, azathioprine or tacrolimus [42-48]. Transplant recipients are indeed known to exhibit an increased incidence of multiple malignancies, encompassing lymphoma as well as hepatocellular carcinoma, Kaposi's sarcoma, and other cutaneous cancers, presumably owing to the state of systemic immunosuppression that is required to avoid rejection [49]. In transplanted patients, rapamycin was associated not only with robust oncosuppressive effects, but also with a *bona fide* anticancer activity against pre-existent tumors, in particular Kaposi's sarcomas [50-54]. Moreover, local or systemic inflammatory responses have been detected in a fraction of transplanted patients on rapamycin-based maintenance regimens [55, 56]. Often, such responses and the consequent toxicity (be it systemic or selectively affecting the transplant) could be promptly reversed by the reintroduction of calcineurin inhibitors [55, 56].

Recently, cancer-preventive and antineoplastic effects have also been attributed to everolimus (also known as RAD001), a rapalog approved by the US FDA for use in patients affected by various malignancies, including renal cell carcinoma (upon the failure of sunitinib- or sorafenib-based chemotherapeutic regimen) [57], subependymal giant cell astrocytoma [58], progressive neuroendocrine tumors of pancreatic origin [59], and advanced estrogen receptor (ER)^+^, *v-erb-b2* avian erythroblastic leukemia viral oncogene homolog 2 (ERBB2)^+^ breast carcinoma (in combination with the aromatase inhibitor exemestane [60]. Although such a beneficial (and completely unsuspected) activity of rapalogs was initially ascribed to their capacity to robustly inhibit the mammalian target of rapamycin (mTOR) complex 1 (mTORC1) in cancer cells, accumulating preclinical evidence indicates that the therapeutic and oncopreventive effects of rapamycin-like compounds originates, at least in part, from cancer cell-extrinsic mechanisms that involve the immune system [61]. In further support of this notion, transplant recipients treated with rapamycin- or everolimus-based maintenance regimens appear to be significantly less predisposed to cytomegalovirus infections than their counterparts receiving conventional immuno-suppressants [62, 63].

Rapamycin and other rapalogs have been shown to exert robust antineoplastic and oncopreventive effects in both transplantable and oncogene-driven tumor models. In immunocompromised mice xenografted with human tumors, this activity obviously reflects cancer cell-intrinsic (or stromal) mechanisms. As a matter of fact, mTORC1 is hyperactivated (hence delivering critical pro-survival signals) in a large number of malignancies, most often due to genetic or epigenetic alterations that result in constitutive signaling via upstream tyrosine kinase receptors (e.g., the epidermal growth factor receptor, EGFR) [64-67]. Conversely, the anticancer effects of rapalogs in immunocompetent settings appear to rely, at least in part, on the elicitation of tumor-targeting immune responses. Rapamycin appears to enhance multiple facets of immune and inflammatory responses elicited in mice by stimuli encompassing replication-competent bacteria [68-73] and viruses [74-77], as well as purified components thereof [74, 78-82] and synthetic immunomodulatory agents [83, 84]. Ovalbumin-specific αβ T lymphocytes exposed to microspheres coated with an ovalbumin-derived peptide plus co-stimulatory CD80 molecules (or with anti-CD3/anti-CD28 antibodies) in the presence of rapamycin exhibited improved memory and antitumor functions *in vivo* than T cells of the same type activated in the presence of interleukin (IL)-12 [85-87]. Along similar lines, rapamycin has been shown to enhance tumor-targeting CD8^+^ T-cell memory responses elicited by a poxviral anticancer vaccine in mice [88, 89]. Such an immunostimulatory activity was observed only when rapamycin was administered in a high-dose short therapeutic course, as opposed to both a single, low-dose course as well as prolonged treatment schedules [88, 89]. Of note, rapamycin has also been shown to increase the yield and effector functions of human γδ T cells activated *in vitro* with isopentenyl pyrophosphate plus recombinant IL-2 [90]. In particular, γδ T cells subjected to antigen stimulation in the presence of rapamycin expressed increased levels of the activation marker CD69, the anti-apoptotic protein BCL-2 and IL-2 receptor α (IL2RA, best known as CD25) [90, 91]. These findings suggest that rapamycin may potentiate purely adaptive immune responses, such as those mediated by αβ T lymphocytes, as well as immune responses with mixed adaptive/innate features, such as those orchestrated by γδ T cells [92, 93]. Other rapalogs, including everolimus and temsirolimus (CCI-779, which has originally been approved by the US FDA for the treatment of advanced renal cell carcinoma in 2007) [94], have been demonstrated to exert immunostimulatory effects, *in vitro* and *in vivo* [77, 95, 96]. As a standalone example, temsirolimus was shown significantly improve the therapeutic potential of a peptide-based anticancer vaccine against established renal cell carcinomas and melanomas in mice [96, 97]. Thus, the potential immunostimulatory activity of rapalogs appears to stem from an on-target effect, i.e., the inhibition of mTORC1 [61, 98, 99].

Taken together, these observations suggest that rapamycin and other rapalogs are capable of stimulating, rather than inhibiting, immune responses, at least under selected circumstances. Whether such an immunostimulatory function truly underlies the anti-aging effects of rapamycin remains to be formally demonstrated. Nonetheless, accumulating preclinical data (Table 1) as well as a large amount of circumstantial clinical evidence (Table 2) suggests that these lifespan-extending chemicals can be harnessed to promote therapeutically relevant antitumor immune responses. Properly designed trials that evaluate the actual immunotherapeutic potential of rapamycin-like compounds are urgently awaited. Alternatively, it will be interesting to see whether circulating or intratumoral biomarkers of pre-existing or therapy-elicited immune responses are capable of identifying a subset of cancer patients that obtain full-blown clinical benefits from the administration of rapalogs. The standardized immuno-monitoring procedures that are required in this context have just begun to be defined and implemented into clinical trials [100, 101]. Altogether, these studies will cast new light on whether rapamycin and other rapalogs should still be considered as immuno-suppressants or whether their immunomodulatory activity, similar that of other drugs like cyclophosphamide [102, 103], rather depends on a large panel of factors, including dose and administration schedule. In this latter scenario, rapalogs may turn out to constitute good candidates for the development of novel immunochemotherapeutic regimens [104].

**Table 1:** Preclinical evidence in support of the immunostimulatory activity of rapalogs

Rapalog	Model	Stimulus	Observation(s)	Ref.
Everolimus (RAD001)	Breast cancer-bearing mice	IL-15-coding plasmid	Improved inhibition of tumor growth	[95]
	HTLV1-infected T cells and patient-derived ATLL cells	IKK inhibitor	Decreased the secretion of IL-10	[77]
	Sprague-Dawley rats	Remnant kidney model	Worsened disease progression correlating with several markers of inflammation	[105]
Sirolimus (Rapamycin)	Human PBMCs and DCs	LPS from *Escherichia coli*	Increased NF-κB activation and pro-inflammatory cytokine secretion; decreased STAT3 activation and IL-10 release	[78]
	Murine DCs	LPS from *Escherichia coli*	Increased secretion of IL-12	[79]
	Monocytes, macrophages and primary DCs	LPS from *Escherichia coli*	Increased NF-κB activation and pro-inflammatory cytokine secretion; decreased STAT3 activation and IL-10 release	[80]
	Murine DCs and C57Bl/10, C3H/HeJ, *Il4ra^−/−^* mice	LPS from *Escherichia coli*	Increased cytokine secretion and improved T-cell co-stimulation	[82]
	Human whole blood	LPS, LTA or peptidoglycan	Inhibition of IL-10 secretion	[81]
	HEK293 cells stably expressing TLR2 of TLR4	*Mycobacterium tuberculosis*	Increased IL-23 secretion at both the mRNA and protein level	[69]
	Murine macrophages, DCs and C57Bl/6 mice	*Mycobacterium tuberculosis*	Enhanced T_H_1 responses in mice vaccinated with sirolimus-treated DCs	[73]
	THP1 cells, primary human PBMCs and DCs	*Staphylococcus aureus*	Increased IL-12 secretion at both the mRNA and protein level	[68]
	Wild-type and transgenic C57Bl/6 mice	*Listeria monocytogenes*	Improved antigen-specific T-cell responses in the course of infection	[70]
	*Traf6^−/−^* mice	Attenuated *Listeria monocytogenes* strain	Improved long-lived CD8^+^ memory T-cell responses	[71]
	DCs from wild-type and PI3K-deficient mice	*Leishmania major*	Improved IL-12 secretion by DCs, robust T_H_1 responses *in vivo*	[72]
	Wild-type and transgenic *Rag1^−/−^* mice	Myxoma virus	Increased anticancer activity of adoptively transferred T lymphocytes	[75]
	Old C57Bl/6 mice	Influenza virus	Improve production of B lymphocytes and optimal responses to vaccination	[76]
	Wild-type and transgenic C57Bl/6 mice	LCMV and engineered vaccinia virus	Increased amounts of antigen-specific T cells	[74]
	HUVECs	Thrombin	Increased NF-κB activation	[106]
	Tumor-bearing transgenic C57Bl/6 mice	Anti-CD3/anti-CD8 antibodies Antigen-derived peptides plus CD80	Generated OT-I cells that were more effective than IL-12-conditioned effector OT-I cells after adoptive transfer	[85]
	Human PBMCs and TU167 cells	IL-2 and isopentenyl pyrophosphate	Increased the yield and effector function of human γδ T cells *in vitro*	[90]
Temsirolimus (CCI-779)	RCC and melanoma-bearing mice	HSP-based anticancer vaccine	Improved CD8^+^ T-cell memory responses and effector functions	[96]

**Abbreviations:** ATLL, adult T-cell leukemia-lymphoma; CAR, chimeric antigen receptor; DC, dendritic cell; HSP, heat-shock protein; HTLV-1, human T-cell lymphotropic virus type 1; HUVEC, human umbilical vein endothelial cell; IKK, IκB kinase; NF-κB, nuclear factor κ-light-chain-enhancer of activated B cells; IL, interleukin; LCMV, lymphocytic choriomeningitis virus; LPS, lipopolysaccharide; LTA, peptidoglycan; PBMC, peripheral blood mononuclear cell; PI3K, phosphoinositide-3-kinase; STAT3, signal transducer and activator of transcription 3; TLR, Toll-like receptor.

**Table 2:** Clinical evidence in support of the immunostimulatory activity of rapalogs

Rapalog	Setting	Observation(s)	Ref.
Everolimus (RAD001)	Cardiac transplantation	Decreased incidence of CMV infection among everolimus-treated patients	[62]
Sirolimus (Rapamycin)	Liver transplantation	Limited rate of HCV progression and associated hepatic fibrosis	[107]
	Renal transplantation	Anemia correlating with biochemical evidence of a chronic inflammatory state	[56]
	Renal transplantation	Development of glomerulonephritis upon conversion from a calcineurin inhibitor-based immunosuppression to rapamycin	[55]
	Solid organ transplantation	Decreased incidence of CMV infection among sirolimus-treated patients	[63]
	Solid organ transplantation	Decreased incidence of multiple tumors among sirolimus-treated patients	[42-48]
	Solid organ transplantation	Consistent antitumor responses in patients with post-transplantation neoplasms treated with sirolimus	[50-54]
Temsirolimus (CCI-779)	Advanced cancer	No signs of immunosuppression among everolimus-treated patients	[108]

**Abbreviations:** CMV, cytomegalovirus; HCV, hepatitis C virus.
